# Trends and Outcomes of Hypothermic Machine Perfusion Preservation of Kidney Allografts in Simultaneous Liver and Kidney Transplantation in the United States

**DOI:** 10.3389/ti.2022.10345

**Published:** 2022-03-14

**Authors:** Alex Chang, Douglas E. Schaubel, Melissa Chen, Peter L. Abt, Therese Bittermann

**Affiliations:** Perelman School of Medicine, University of Pennsylvania, Philadelphia, PA, United States

**Keywords:** graft survival, delayed graft function, simultaneous liver-kidney transplantation, allograft preservation, allograft outcomes, primary non-function, center variability

## Abstract

Optimal kidney graft outcomes after simultaneous liver-kidney (SLK) transplant may be threatened by the increased cold ischemia time and hemodynamic perturbations of dual organ transplantation. Hypothermic machine perfusion (MP) of kidney allografts may mitigate these effects. We analyzed U.S. trends and renal outcomes of hypothermic non-oxygenated MP vs. static cold storage (CS) of kidney grafts from 6,689 SLK transplants performed between 2005 and 2020 using the United Network for Organ Sharing database. Outcomes included delayed graft function (DGF), primary non-function (PNF), and kidney graft survival (GS). Overall, 17.2% of kidney allografts were placed on MP. Kidney cold ischemia time was longer in the MP group (median 12.8 vs. 10.0 h; *p* < 0.001). Nationally, MP utilization in SLK increased from <3% in 2005 to >25% by 2019. Center preference was the primary determinant of whether a graft underwent MP vs. CS (intraclass correlation coefficient 65.0%). MP reduced DGF (adjusted OR 0.74; *p* = 0.008), but not PNF (*p* = 0.637). Improved GS with MP was only observed with Kidney Donor Profile Index <20% (HR 0.71; *p* = 0.030). Kidney MP has increased significantly in SLK in the U.S. in a heterogeneous manner and with variable short-term benefits. Additional studies are needed to determine the ideal utilization for MP in SLK.

## Graphical Abstract

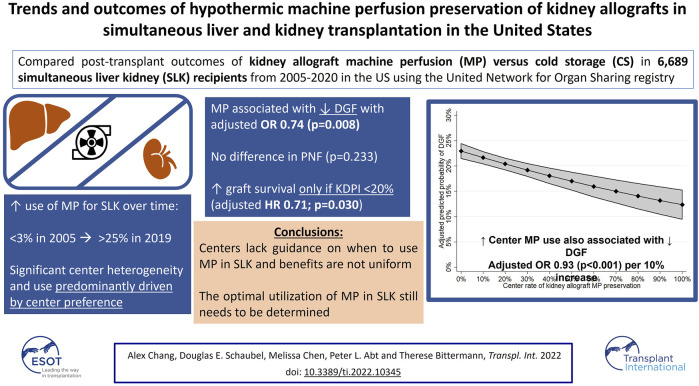

## Introduction

Outcomes after orthotopic liver transplantation (LT) are strongly associated with pre- and post-operative renal failure, and patient survival is significantly lower in recipients requiring long-term dialysis post-transplant (1). Thus, it is widely accepted that selected patients with pre-LT renal dysfunction be considered for simultaneous liver-kidney transplant (SLK) to improve their outcomes after LT (2). In the years following the introduction of the Model for End-stage Liver Disease (MELD) allocation system in 2002, the rate of SLKs increased dramatically (3). This partly resulted from an organ allocation system that prioritized candidates with worse renal function and from the implementation of policies that facilitated access to SLK. According to the most recently published national data, 7.1% of candidates on the LT waitlist were awaiting SLK and 8.6% of completed LTs were performed with a concurrent kidney transplant (KT) in 2018 (4). However, despite being of higher quality, kidney graft survival after SLK has been shown to be worse than after KT alone, particularly in the early post-LT period, which has been primarily attributed to the greater severity of illness of SLK recipients (5, 6).

Machine perfusion (MP) of deceased donor kidney grafts has been used as an alternative to static cold storage (CS) as a means to improve post-transplant kidney function, particularly for allografts of reduced quality (7). After the allograft is flushed free of blood, MP pumps hypo- or normothermic preservation solution through the renal vasculature in a manner that simulates natural organ perfusion, leading to clearance of toxic metabolites and reduced renovascular resistance (8). While MP has primarily been used in the setting of marginal kidney allografts for KT alone, a recent observational study by Lunsford et al. conducted at two U.S. transplant centers has suggested that MP may also improve kidney graft outcomes among SLK recipients (9).

Given these recent findings, we sought to evaluate 1) temporal and geographic changes in the use of kidney graft MP preservation in SLK and 2) evaluate the potential benefit of MP on patient and kidney graft outcomes in a national cohort.

## Methods

This was a retrospective cohort study using the United Network for Organ Sharing (UNOS) database. All adult (≥18 years), deceased-donor simultaneous liver-kidney (SLK) transplant recipients between January 1, 2005 and December 6, 2020 were identified. Recipients of prior solid organ transplant of any kind were excluded. Status 1 (i.e., emergent LT) recipients were additionally excluded.

The primary exposure of interest was receipt of a kidney allograft preserved using MP versus CS. Given the focus of the study, all analyses were restricted to SLK recipients for whom kidney allograft preservation data was available (98.8% of the initial cohort). While detailed information regarding MP protocols used was not available (e.g., duration, flow, resistance), it should be noted that all currently approved devices by the US Food and Drug Administration are hypothermic non-oxygenated systems. Recipient characteristics obtained at the time of SLK included: age, sex, race/ethnicity, kidney disease etiology, history of diabetes, native Model for End-stage Liver Disease (MELD), cirrhosis decompensations (ascites, hepatic encephalopathy), patient location prior to SLK (home, inpatient ward, intensive care unit), severity of renal disease at SLK (on dialysis, eGFR <30 ml/min/1.73 m^2^ not on dialysis and eGFR ≥30 ml/min/1.73 m^2^ not on dialysis), and duration of dialysis (among those on dialysis at SLK). Donor characteristics included: age, sex, race/ethnicity, hypertension, diabetes, body mass index (BMI), terminal creatinine, hepatitis C virus (HCV) antibody status, distance from recipient hospital and cause of death (COD). Additional allograft characteristics included donation after circulatory determination of death status (DCD), cold ischemic time (CIT), whether liver allograft was split, Kidney Donor Profile Index (KDPI; categorized as <20%, 30–34%, 35–85%, and >85% (10, 11)) and share type (local, regional, national). Lastly, we also evaluated whether kidney implantation occurred on the same versus ≥1 day after the date of LT.

Recipient, donor and allograft characteristics were compared descriptively according to preservation using MP versus CS. Chi-squared tests and Kruskal-Wallis tests were used for categorical and continuous variables, respectively. Temporal, regional and center trends in MP use were also described. The geographic distribution of the 11 UNOS regions can be visualized for reference here: https://unos.org/community/regions/. In the first analysis, mixed-effects multivariable logistic regression was employed to evaluate the predictors of MP kidney allograft preservation. This model was adjusted for the aforementioned exposures as fixed effects (with the exception of KDPI to avoid collinearity, as the individual index components were already included) and transplant center as a random effect. From this model, the intraclass correlation coefficient (ICC) was obtained, which indicates the percent variability in MP perfusion across recipients that is explained by transplant center alone.

All subsequent statistical analyses evaluated receipt of MP as a predictor of recipient outcomes. Mixed-effects multivariable logistic regression was used to investigate kidney delayed graft function (DGF) and 2) kidney allograft primary non-function (PNF). Adjustment covariates included each of the aforementioned recipient and donor/allograft characteristics (except KDPI), as well as transplant era (2005–2009, 2010–2014, 2015–2020). All covariates were represented by fixed effect, with the exception of transplant center which was specified as a random effect in order to efficiently account for correlation among patients within-center. DGF was defined as receipt of dialysis within the first week after SLK (12, 13). PNF was defined as kidney graft failure ≤90 days from the date of SLK (14). Kidney graft survival was the time between transplantation and the earliest of retransplantation or death.

DGF was modeled using mixed logistic regression using all of the above-listed adjustment covariates and a random center effect. In the multivariable model investigating PNF, a parsimonious model was developed given the low number of events (a total of 124 patients experienced PNF). Stepwise forwards selection with *p*-value thresholds of <0.05 and ≥0.1 for entry and removal, respectively, was used to select covariates for the final model. Cox regression was used to model graft survival. Analogous to DGF, all of the adjustment covariates were included, with center again represented through a random effect.

After fitting each of the above-described models, we evaluated interactions with kidney allograft perfusion strategy. To evaluate the interactions with MP, we adopted the same general strategy for each of the tree outcomes. In particular, all main effects remained in the model. First, we evaluated each interaction separately one at a time. Second, any significant interactions would then be evaluated simultaneously to avoid confounding. In order to ensure clinical interpretability of our findings, we restricted attention to a pre-specified set of covariates for which interaction with MP was felt by the investigators to have biological plausibility. This set included each of the KDPI components (i.e., donor age, race/ethnicity, BMI, history of hypertension, history of diabetes, cause of death, terminal creatinine, HCV antibody status, and DCD status (15)), KDPI (categorized as <20%, 30–34%, 35–85%, and >85% (10, 11)), renal allograft CIT (continuous) and recipient renal disease severity (on dialysis, eGFR <30 ml/min/1.73 m^2^ not on dialysis and eGFR ≥30 ml/min/1.73 m^2^ not on dialysis). In models evaluating the interaction of MP and KDPI, the individual components of the KDPI were not included given concern for collinearity and lack of interpretability. Note that, for PNF, we excluded covariates not chosen earlier (for the main effects model) from the above list of potential interaction variables.

Next, we carried out secondary analyses. First, we evaluated unadjusted rates of each outcome according to whether kidney implantation was delayed or not among those undergoing MP preservation using descriptive statistics. Second, we replaced the (patient-level) MP indicator with center-level percentage of patients transplanted with a perfused kidney. This equates to changing the question posed from “what is the effect of MP on patients” outcome in the primary analyses to “what is the effect of a center using more kidney MP on patients” outcome (i.e., irrespective of type of kidney perfusion strategy received). Center MP rate was evaluated as a predictor of each of the three outcomes (DGF, PNF, kidney graft survival) without adjusting for center (since doing so is inappropriate in the presence of center-level covariates). For the models evaluating DGF and kidney graft survival, the final multivariable model adjusted for all covariates. For the model evaluating PNF, the same covariate selection method described previously was used, which selected the same covariates as in the primary multivariable model.

All analyses were performed using STATA v16 (College Station, TX, United States). This study was approved by the Institutional Review Board of the University of Pennsylvania.

## Results

There were 6,689 recipients of SLK between January 1, 2005 and December 6, 2020. Allograft storage type (i.e., MP vs. CS) was available in 6,610 (98.8%) recipients. Of these, 5,474 (82.8%) kidney allografts for SLK underwent static CS, while 1,136 (17.2%) received MP preservation.

**FIGURE 1:**
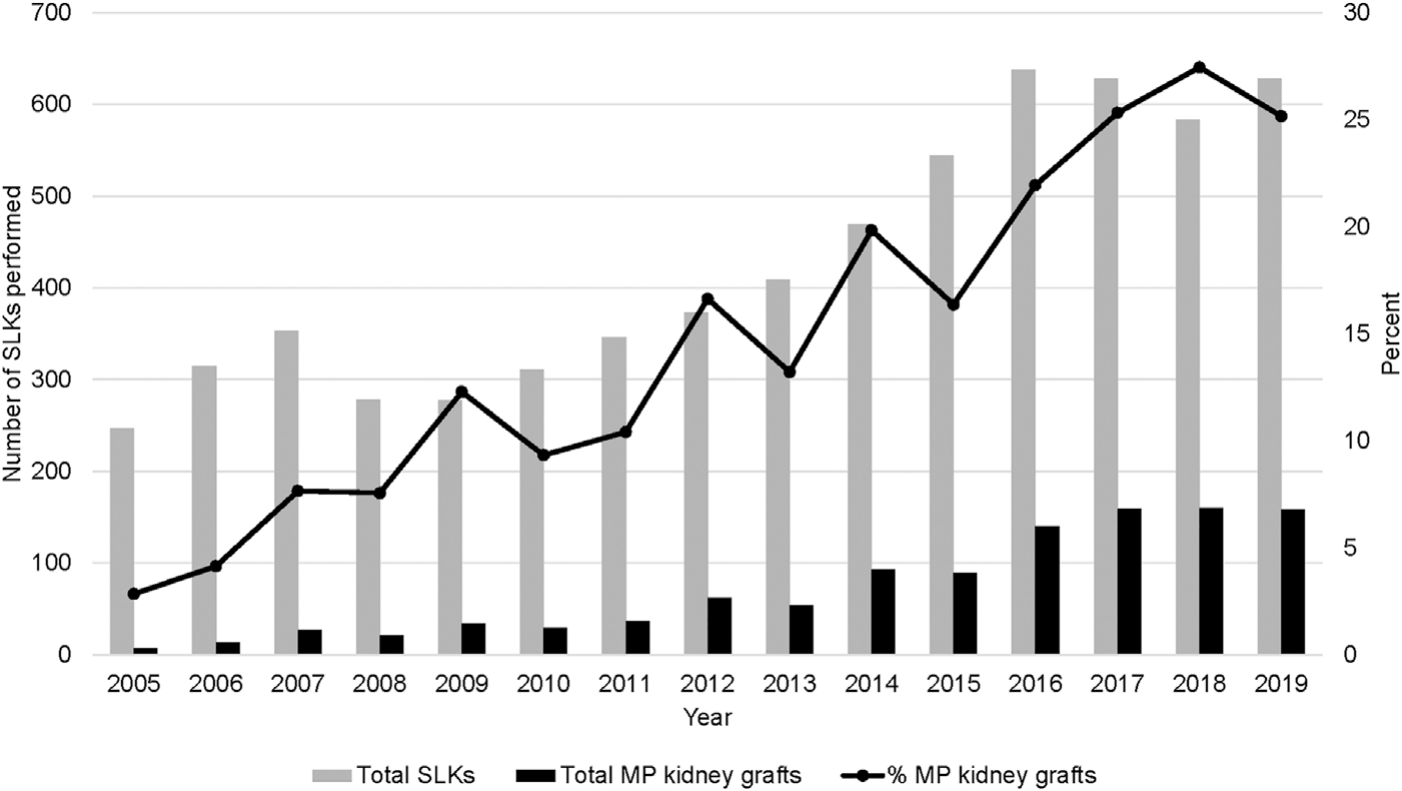
Nationwide trends in MP use in SLK transplants from 2005–2019.

**FIGURE 2:**
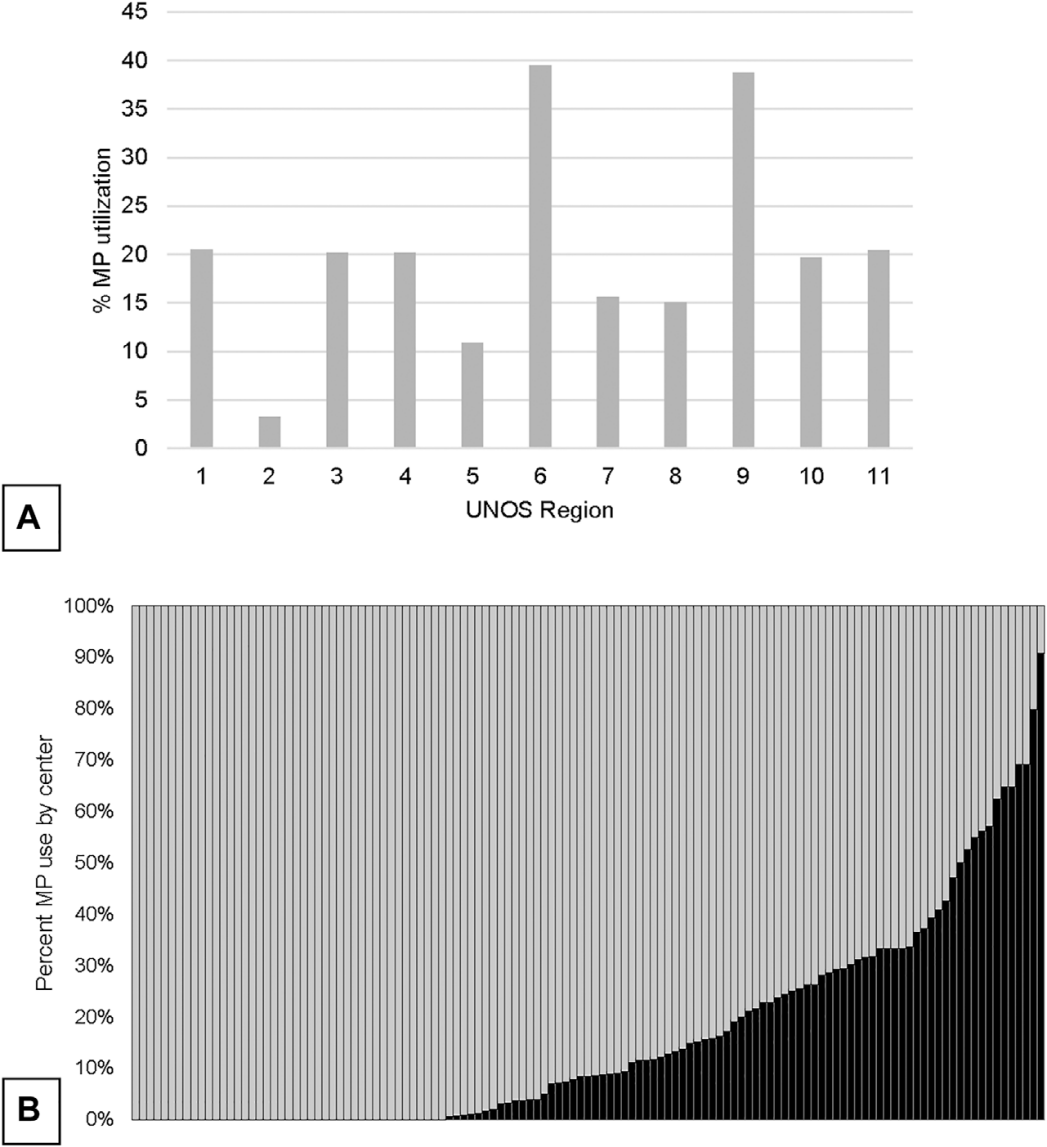
Variation in overall utilization of MP in SLK by UNOS region **(A)** and by center **(B)**.

Concurrent to the increase in SLK volume between 2005 and 2018, the utilization of kidney allograft MP also increased from 2.8% in 2005 to 25.2% in 2019 (Figure 1). There was significant geographic variability in the utilization of MP for SLK between UNOS regions and individual transplant centers (Figure 2). UNOS region two had the lowest utilization, with 3.3% of 668 SLKs between 2005 and 2020, while region six had the highest with 39.5% of 119 SLKs. Of the 125 centers included in the analysis, 34.4% (N = 43 centers) exclusively used CS in SLK. MP use at the remaining 82 centers ranged from 0.6% to 90.9%. There was no correlation between center MP use and center SLK volume (*p* = 0.131), median KDPI (*p* = 0.743) or median SLK waiting time (*p* = 0.455).

### Donor and Recipient Characteristics According to Kidney Allograft Preservation Technique

Donors whose kidneys underwent MP were older than those undergoing CS: median 36 (IQR: 24–47) versus 34 (IQR: 26–49) years (*p* < 0.001, Table 1), though this difference was small. They were also more likely to have diabetes: 6.3% versus 4.2% (*p* = 0.002). There was no statistically significant difference in donor sex, race/ethnicity, terminal creatinine, or HCV antibody status between allografts preserved using MP versus CS. Kidney allografts undergoing MP were more often DCD organs (7.9% vs. 4.5%, *p* < 0.001) and had longer CIT (median 12.8 vs. 10.0 h, *p* < 0.001). Of note, there was no statistical difference in liver allograft CIT between groups (*p* = 0.074). A trend towards higher KDPI among recipients of MP preserved kidney allografts was noted (*p* = 0.003; Table 1).

**TABLE 1:** Donor characteristics according to kidney allograft preservation technique (N = 6,610).

	Cold preservation	Machine perfusion	*p*-value
	N = 5,474	N = 1,136	
Sex, N (%)			0.660
Male	3,388 (61.9)	711 (62.6)	
Female	2,086 (38.1)	425 (37.4)	
Age (years), median (IQR)	34 (24–47)	36 (26–49)	<0.001
Race/ethnicity, N (%)			0.341
White	3,569 (62.5)	750 (66.0)	
Black	849 (15.5)	169 (14.9)	
Hispanic	836 (15.3)	183 (16.1)	
Asian/other	220 (4.0)	34 (3.0)	
Hypertension, N (%)	1,153 (21.2)	264 (23.5)	0.091
Diabetes, N (%)	228 (4.2)	71 (6.3)	0.002
KDPI category, N (%)			0.003
<20%	1,933 (36.5)	356 (31.4)	
20–34%	1,022 (18.7)	237 (20.9)	
35–85%	2,235 (40.9)	482 (42.5)	
>85%	210 (3.9)	59 (5.2)	
DCD donor, N (%)	224 (4.5)	90 (7.9)	<0.001
Kidney CIT (hours), median (IQR)	10.0 (7.7–12.8)	12.8 (9.4–21.7)	<0.001
Liver CIT (hours), median (IQR)	6.1 (5.0–7.7)	6.0 (4.7–7.6)	0.074
Split liver, N (%)	81 (1.5)	12 (1.1)	0.270
Distance to donor (miles), median (IQR)	59 (8–158)	52 (8–166)	0.492
Share type, N (%)			0.018
Local	4,099 (74.9)	858 (75.5)	
Regional	1,241 (22.7)	235 (20.7)	
National	134 (2.5)	43 (3.8)	
Cause of death, N (%)			0.002
Anoxia	1,625 (29.7)	407 (35.8)	
Stroke	1,494 (27.3)	277 (24.4)	
Head trauma	2,188 (40.0)	420 (37.0)	
CNS tumor	35 (0.64)	5 (0.4)	
Other	132 (2.4)	27 (2.4)	
BMI (kg/m^2^), median (IQR)	25.8 (22.8–29.8)	26.4 (23.3–30.1)	0.001
Terminal creatinine (mg/dl), median (IQR)	0.9 (0.7–1.2)	0.9 (0.7–1.2)	0.267
HCV antibody positive, N (%)	399 (7.3)	88 (7.8)	0.599

Few recipient characteristics were associated with kidney allograft MP versus CS preservation (Table 2). For example, no statistically significant differences were observed with regards to age, sex, or native MELD score. While statistically significant, differences in cirrhosis decompensations such as ascites severity or hepatic encephalopathy grade were clinically less relevant, as they were very small (*p* < 0.001 and *p* = 0.018, respectively). There was no statistically significant difference in pre-LT renal disease severity between groups (*p* = 0.458). However, among recipients on dialysis pre-SLK (N = 4,590), pre-transplant dialysis duration was longer for patients receiving allografts preserved using MP: median 6.1 months versus 3.7 months (*p* < 0.001). Etiology of kidney disease was also different (*p* < 0.001) with those having hepatorenal syndrome receiving MP kidney grafts more frequently than those with cold storage (40.9% vs. 30.2%). Kidney implantation occurred ≥1 day after LT for 34.9% of patients in the MP group versus 13.9% in the CS group (*p* < 0.001).

**TABLE 2:** Recipient characteristics at LT according to donor kidney allograft preservation technique (N = 6,610).

	Cold storage	Machine perfusion	*p*-value
	N = 5,474	N = 1,136	
Sex, N (%)			0.324
Male	3,482 (63.6)	705 (62.1)	
Female	1,992 (36.4)	431 (37.9)	
Age (years), median (IQR)	58 (51–63)	58 (52–64)	0.222
Race/ethnicity			0.072
White	3,386 (61.9)	724 (63.7)	
Black	807 (14.7)	158 (13.9)	
Hispanic	992 (18.1)	196 (17.3)	
Asian	211 (3.9)	32 (2.8)	
Other	78 (1.4)	26 (2.3)	
Native MELD at SLK, median (IQR)	28 (23–35)	28 (23–35)	0.457
Ascites, N (%)			<0.001
None	885 (16.2)	230 (20.3)	
Mild	2,182 (40.1)	385 (34.0)	
Moderate-severe	2,381 (43.7)	519 (45.8)	
Encephalopathy, N (%)			0.018
None	1,697 (31.2)	401 (35.4)	
Grade 1–2	2,991 (54.9)	577 (50.9)	
Grade 3–4	760 (14.0)	156 (13.8)	
Preop location, N (%)			0.514
Home	3,142 (57.5)	674 (59.4)	
Inpatient ward	1,311 (24.0)	262 (23.1)	
ICU	1,008 (18.5)	199 (17.5)	
Diabetes, N (%)	2,356 (43.3)	488 (43.2)	0.905
Kidney disease severity, N (%)			
eGFR ≥30 ml/min/1.73 m^2,^ ^a^	677 (12.7)	157 (14.1)	0.458
eGFR <30 ml/min/1.73 m^2,^ ^a^	1,445 (27.1)	295 (26.4)	
On dialysis	3,213 (60.2)	665 (59.5)	
Dialysis time^b^ (months), median (IQR)	3.7 (0.9–14.9)	6.1 (1.5–21.5)	<0.001
Etiology of kidney disease, N (%)			<0.001
Hepatorenal syndrome	1,655 (30.2)	465 (40.9)	
Diabetes	1,134 (20.7)	225 (19.8)	
Glomerular disease	426 (7.8)	78 (6.9)	
Polycystic kidney disease	278 (5.1)	88 (7.8)	
Hypertension	476 (8.7)	74 (6.5)	
Other	1,505 (27.5)	206 (18.1)	
KT implantation ≥1 day after LT, N (%)	760 (13.9)	396 (34.9)	<0.001

a Not on dialysis pre-LT.

b Among patients receiving dialysis prior to SLK (N = 4,590).

### Predictors of Kidney Allograft MP Preservation in SLK

In adjusted analyses, several predictors of kidney allograft MP preservation were identified (Supplementary Table S1). These included: increasing donor age (OR 1.02 per 1 year increase, 95% CI: 1.01–1.03, *p* < 0.001), DCD status (OR 2.81, 95% CI: 1.88–4.20; *p* < 0.001), kidney allograft CIT (1.10 per 1 h increase, 95% CI: 1.08–1.11; *p* < 0.001), donor terminal creatinine (OR 1.22 per 1 mg/dl increase, 95% CI: 1.08–1.39; *p* = 0.001), and donor BMI (OR 1.02 per 1 kg/m^2^ increase; 95% CI 1.00–1.04; *p* = 0.020). Regionally shared kidney allografts were associated with less use of MP preservation (OR 0.47 vs. local, 95% CI: 0.36–0.61; *p* < 0.001). Transplant era was strongly associated with MP use: OR 2.42 (95% CI: 1.72–3.39) for 2010–2014 and OR 6.03 (4.30–8.44) for 2015–2020 versus 2005–2009 (*p* < 0.001). The ICC for transplant center in this model was 65.0%. This indicates that nearly two-thirds of the variability in MP use across SLK recipients was explained by the transplanting center alone, while donor and recipient factors explained only a minority.

### Kidney Allograft Preservation Technique and Delayed Graft Function

DGF occurred in 256 recipients after MP and 1,311 recipients after CS (22.5% vs. 24.0%, *p* = 0.293). There was no statistical difference in DGF rates among MP allografts implanted on the same versus on a subsequent date from LT (22.0% vs. 23.6%; *p* = 0.554). Accounting for recipient and donor covariates, transplant era and transplant center, MP was significantly associated with DGF in the final multivariable model with a covariate-adjusted OR of 0.74 (95% CI: 0.60–0.92; *p* = 0.008; Table 3). The results of the full multivariable model are shown in Supplementary Table S2. There were no statistically significant interactions found between kidney allograft preservation type and any of the covariates evaluated.

**TABLE 3:** Summary of findings obtained from multivariable models evaluating kidney allograft preservation type as a predictor of kidney graft outcomes after SLK.

	Point estimate (95%CI) for kidney allograft MP compared to CS	*p*-value
Kidney delayed graft function	OR 0.74 (0.60–0.92)	0.008
Kidney primary non-function	OR 0.88 (0.52–1.49)	0.637
Kidney graft survival	HR 0.91 (0.78–1.06)	0.230

**FIGURE 3:**
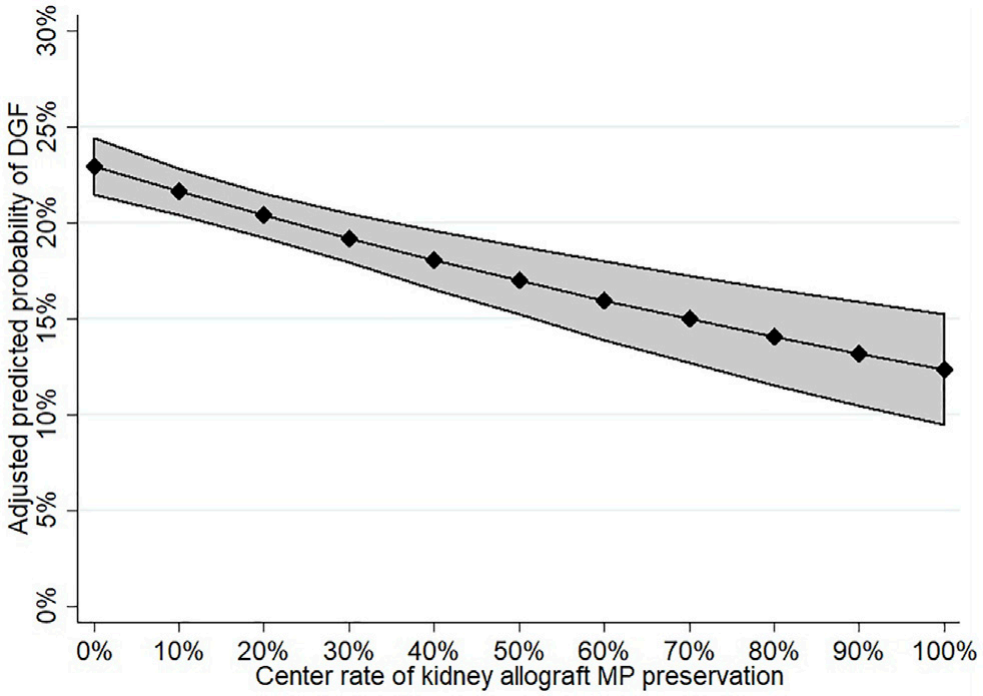
Adjusted predicted probability of DGF according to increasing center MP use.

As a secondary analysis, center kidney allograft MP use was evaluated as an independent predictor of recipient DGF. Center practice was found to be associated with a reduction in the odds of DGF in both univariable (OR 0.94 per 10% increase in center MP use, 95% CI: 0.92–0.97; *p* < 0.001) and multivariable analyses (OR 0.93 per 10% increase in MP use, 95% CI: 0.90–0.96; *p* < 0.001; Supplementary Table S3). The predictive margins of DGF by increasing center kidney allograft MP are shown in Figure 3.

### Kidney Allograft Preservation Technique and Primary Non-function

Kidney allograft PNF occurred in 19 patients after MP and 105 patients after CS (1.9% vs. 2.1%, *p* = 0.666). There was no difference in PNF rate for MP kidneys with delayed implantation (2.0% vs. 1.9%; *p* = 0.849). MP was not associated with PNF in the final multivariable model: covariate-adjusted OR 0.88 (95% CI: 0.52–1.49; *p* = 0.637; Supplementary Table S4). No statistically significant interaction was found between MP use and any of the covariates studied, which included recipient renal disease severity, kidney donor KDPI, donor age, donor BMI, donor hypertension, donor cause of death or kidney allograft CIT. In secondary analyses, center MP use was not associated with kidney allograft PNF on either univariable (OR 0.94 per 10% increase in MP use, 95% CI: 0.86–1.03; *p* = 0.180) or multivariable analyses (OR 0.94, 95% CI: 0.85–1.04; *p* = 0.233; Supplementary Table S5).

### Kidney Allograft Preservation Technique and Kidney Allograft Survival

**FIGURE 4:**
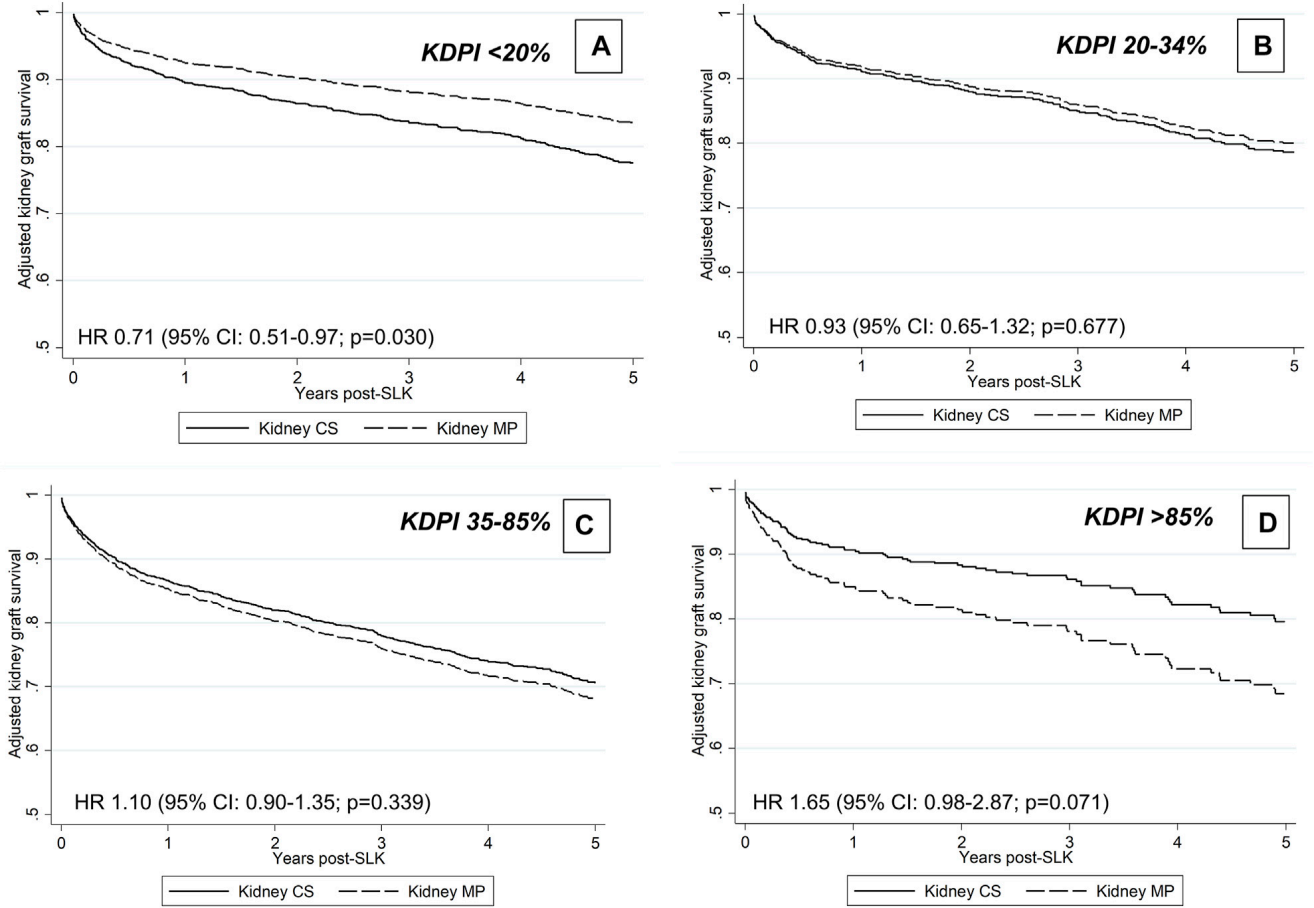
Adjusted graft survival associated with MP preservation according to KDPI group.

Kidney allograft MP was not associated with unadjusted or adjusted kidney graft survival, defined as a combined end-point of kidney graft failure or patient death: HR 0.95 (95% CI: 0.82–1.10, *p* = 0.481) and HR 0.91 (95% CI: 0.76–1.03; *p* = 0.230; Supplementary Table S6), respectively. Of the covariates evaluated for interaction with kidney allograft perfusion type, the following were statistically significant: donor KDPI category (*p* = 0.029) and donor cause of death (*p* = 0.039). The results of the multivariable model including the interaction of perfusion type and KDPI category are shown in Supplementary Table S7. In stratified models by KDPI category, MP was associated with improved graft survival in the setting of KDPI <20% (adjusted HR 0.71, 95% CI: 0.53–0.97; *p* = 0.030, but not with higher KDPI (*p* = 0.677 for KDPI 20–34%, *p* = 0.339 for 35–85% and *p* = 0.071 for >85%; Figure 4). Unfortunately, the interaction between perfusion type and donor cause of death was entirely driven by the “other” category, which is clinically uninterpretable and, thus, not included in the final model. Center MP use was not associated with kidney graft survival in multivariable analyses: covariate-adjusted HR 1.00 (95% CI: 0.98–1.03; *p* = 0.851; Supplementary Table S8). In unadjusted analyses, there was no improvement in kidney graft survival among MP allografts with delayed implantation (log-rank *p* = 0.741).

## Discussion

In this analysis of national data over a 15-year period, we show that the use of MP preservation in SLK has markedly increased in the U.S. over time, accounting for 1 in 4 kidney allografts since 2017. Several studies have shown benefits of MP preservation compared to static CS in the setting of KT alone (16–18). However, its potential benefits have not been rigorously studied in SLK transplantation until now, a scenario in which 1) increased kidney allograft quality and 2) the added complexity of dual-organ transplantation may reduce the advantages of MP preservation. In this study, we find a significant reduction in kidney DGF with MP preservation and increased center MP utilization also predicted lower DGF. In contrast, we found no association between kidney allograft MP and PNF, and only benefits with respect to kidney graft survival among the highest quality kidney allografts. The present study additionally demonstrates large practice variability between transplant centers in the choice of kidney allograft preservation modality in SLK. In fact, where one undergoes SLK explained the majority of the variability in kidney allograft MP use, while the 25 other donor and recipient factors were lesser determinants. It is likely that anecdotal experience and the existing evidence-base in the KT alone population has driven the rapid expansion of MP preservation for SLKs at these centers. However, further studies are needed to more clearly delineate which SLK recipients stand most to benefit from the added resources and associated costs of kidney MP preservation.

It is well-established that SLK recipients have access to the highest quality kidney allografts (19). In several meta-analyses of clinical trials, MP preservation in KT alone has benefits with regards to short- and long-term graft outcomes. This has not only been demonstrated in marginal donor kidneys but also in standard quality organs (7, 20). This technique has also been shown to be more cost-effective over CS, irrespective of kidney graft quality (21). Yet, perhaps surprisingly, in the SLK population, we only found evidence of reduced DGF and limited improvements in long-term graft outcomes, despite accounting for other measures of inferior allograft quality in our analyses. Nevertheless, the significant reduction in DGF in this population should not be overlooked, particularly given the high quality of kidney allografts allocated to SLK recipients.

Recipient factors play important and unique roles in the development of poor kidney graft outcomes in the SLK population. These include, among others, increased liver disease severity, intra-operative challenges (e.g., volume shifts, transfusion requirements, electrolyte disturbances) and prolonged post-transplant recovery than KT alone recipients. The significant contribution of recipient factors on graft outcomes may explain why the benefits of MP were only observed in those receiving allografts with KDPI <20%, which represented 35.5% of the cohort. Delayed kidney implantation (as evidenced by the longer kidney CIT and difference in transplant dates recorded) was more frequent in the MP group. However, we did not observe any differences in unadjusted graft outcomes according to timing of kidney implantation in the MP group. Thus, the proposed benefits of MP preservation to allow for delayed KT in a more optimal recipient milieu after LT remain uncertain.

Our results using national data differ from those published by others reporting their own center-specific experiences, in which MP preservation with delayed KT implantation offered clear superior results, including resultant effects on patient survival (9, 22). These differences in findings are likely partly explained by the association between increasing center preference for MP and the associated reduction in DGF identified in this study, as centers with established MP protocols are more likely to publish on their experiences compared to those that seldom use MP. In addition, while we were able to determine type of preservation modality and duration of CIT, whether centers and organ procurement organizations (OPOs) differed with respect to the proportion of time spent on pump, time from procurement to placement on pump, other aspects of MP-related management and decision-making regarding potential delayed timing of implantation were not known. This may also explain why smaller gains were observed with kidney allograft MP preservation when this practice was evaluated nationally, and which would highlight the need for more clearly defined “best practices” regarding when and how to employ MP preservation to maximize its impact on kidney graft outcomes in SLK. Further research using more comprehensive donor data and allograft quality indicators, such as that collected from OPOs, may provide greater insights into the ideal setting to use MP preservation in SLK.

While the use of a national cohort offers advantages, there are also inherent study limitations. All commercially available MP devices for kidney allografts in the US are hypothermic non-oxygenated systems. However, more granular data regarding the duration of pumping and other MP parameters (e.g., flow, resistance) were not available and likely varied by center and OPO. This may have biased certain results towards the null. In addition, while we were able to examine common recipient and allograft predictors of MP use, more comprehensive details on centers’ decision-making and protocols are not known. Similarly, there may be differences regarding kidney allograft management that occurred at the OPO-level before the organ arrived at the transplanting center. If heterogeneity in MP protocol is indeed the explanation for the null result obtained in our study, then this speaks to the need for greater evidence-based guidance on its use and further multi-center studies are warranted that could address this evidence gap. The relationship observed between increasing center MP use and declining DGF rates may support the notion that centers with more MP experience use this technology more effectively and thus a “learning curve” for MP exists, which may further contribute to the outcomes seen.

Other limitations of registry data include diminished donor and recipient clinical detail. This could have led to unmeasured confounding and subsequent bias in our results. There were also no recipient peri- or post-operative clinical details between transplant surgeries to confirm that the longer kidney CIT and differences in KT versus LT transplant dates recorded for the MP group indeed reflected the intention to delay kidney implantation to allow for a more favorable recipient clinical status. Supporting this is the fact that indicators of kidney allograft quality and recipient factors explained only a minority of the variability in MP use across centers, and thus this decision-making infrequently takes into account key variables known to be associated with inferior kidney graft outcomes (23–25). Given the available variables, geographic trends analysis was limited to UNOS regions. This issue should be re-evaluated in the future, particularly in the context of the new liver allocation system in the U.S, which has led to greater transportation of allografts (26). Lastly, the imbalance between the MP and CS sample sizes may have led to imprecision in the point estimates and the adjustment of measured confounders in the multivariable models. In particular, given the low number of PNF events particularly among MP patients, it is likely that power was inadequate to detect a significant difference. Moreover, given the low frequency of high KDPI kidneys in this SLK cohort, a potential difference in graft survival with MP may have been missed.

A rapidly increasing use of MP for storage of kidney allografts prior to SLK transplantation has occurred in the U.S. that is predominantly driven by transplant center preference. While MP kidney allograft preservation affords a reduction in DGF, its impact on longer-term outcomes for the majority of recipients remain uncertain. There is a need to understand the cost-effectiveness and logistical implications of this increasing MP use (with or without kidney implantation delay), and more comprehensive guidance is also warranted with respect to when and how to best use this potentially valuable technology in the SLK population.

## Data Availability

Publicly available datasets were analyzed in this study. This data can be found here: https://unos.org/data/.

## Abbreviations

BMI, body mass index; CIT, cold ischemia time; COD, cause of death; CS, cold storage; DCD, donation after circulatory death; HCV, hepatitis C virus; HLA, human leukocyte antigen; KDPI, kidney donor profile index; LT, liver transplant; MELD, Model for End Stage Liver Disease; MP, machine perfusion; OPO, organ procurement organization; OPTN, Organ Procurement and Transplantation Network; PNF, primary non-function; SLK, simultaneous liver and kidney; UNOS, United Network for Organ Sharing.
