# Supplementary material for: Trends and Outcomes of Hypothermic Machine Perfusion Preservation of Kidney Allografts in Simultaneous Liver and Kidney Transplantation in the United States
Source: Transpl Int. 2022 Mar 14;35:10345. doi: 10.3389/ti.2022.10345 (PMC8958417; doi:10.3389/ti.2022.10345)
Supplement: Supplementary file 1 [file DataSheet1.pdf]

Supplemental Table 1: Mixed-effects multivariable model evaluating the recipient and donor predictors of MP preservation in SLK (N=5,988)

	Covariate-adjusted OR (95% CI)	p-value
Recipient female sex	1.20 (0.98-1.47)	0.072
Recipient age, per year	1.00 (0.99-1.01)	0.590
Recipient race/ethnicity		0.760
White	Reference	
Black	0.86 (0.64-1.14)	
Hispanic	0.88 (0.66-1.19)	
Asian	0.82 (0.48-1.40)	
Other	1.04 (0.56-1.95)	
Native MELD at LT, per point	1.01 (0.99-1.03)	0.359
Kidney disease severity		0.826
On dialysis	Reference	
eGFR <30mL/min/1.73m^2*	0.94 (0.75-1.17)	
eGFR ≥30mL/min/1.73m^2*	1.02 (0.75-1.38)	
Ascites		0.503
None	Reference	
Mild	0.84 (0.62-1.13)	
Moderate-severe	0.89 (0.65-1.20)	
Encephalopathy		0.290
None	Reference	
Grade 1-2	1.18 (0.92-1.51)	
Grade 3-4	0.98 (0.68-1.41)	
Recipient location		0.961
Home	Reference	
Inpatient	1.00 (0.76-1.31)	
ICU	1.04 (0.75-1.45)	
Recipient diabetes	1.02 (0.81-1.29)	0.867
Recipient kidney disease		0.525
Glomerular disease	Reference	
Diabetes	1.02 (0.66-1.56)	
Hypertension	0.92 (0.55-1.51)	
Polycystic kidney disease	1.22 (0.73-2.04)	
Hepatorenal syndrome	0.83 (0.56-1.22)	
Other	0.94 (0.63-1.40)	
Donor age, per year	1.02 (1.01-1.03)	<0.001
Female donor sex	0.99 (0.81-1.21)	0.911
Donor race/ethnicity		0.249
White	Reference	
Black	1.16 (0.89-1.53)	
Hispanic	1.21 (0.91-1.63)	
Asian/other	0.74 (0.43-1.28)	
Donor hypertension	1.04 (0.81-1.34)	0.753
Donor diabetes	1.47 (0.96-2.24)	0.073
DCD donor	2.81 (1.88-4.20)	<0.001
Kidney graft CIT, per hour	1.10 (1.08-1.11)	<0.001

Split liver	0.74 (0.32-1.74)	0.488
Share type		<0.001
Local	Reference	
Regional	0.47 (0.36-0.61)	
National	0.67 (0.40-1.13)	
Donor HCV Ab positive	1.12 (0.77-1.63)	0.555
Donor terminal creatinine, per 1mg/dL	1.22 (1.08-1.39)	0.001
Donor BMI, per kg/m^2	1.02 (1.00-1.04)	0.020
Donor cause of death		0.359
Anoxia	Reference	
CVA	0.77 (0.58-1.01)	
Head trauma	0.98 (0.77-1.24)	
CNS tumor	0.64 (0.19-2.18)	
Other	0.83 (0.45-1.52)	
Transplant era		<0.001
2005-2009	Reference	
2010-2014	2.42 (1.72-3.39)	
2015-2020	6.03 (4.30-8.44)	

Supplemental Table 2: Mixed-effects multivariable logistic regression model evaluating kidney allograft preservation strategy as a predictor of delayed graft function (N=5,986)

	Covariate-adjusted OR for DGF (95% CI)	p-value
**Kidney allograft preservation**		**0.008**
**Cold storage**	**Reference**	
**Machine perfusion**	**0.74 (0.60-0.92)**	
Recipient female sex	1.10 (0.96-1.27)	0.180
Recipient age, per year	1.00 (0.99-1.00)	0.423
Recipient race/ethnicity		0.091
White	Reference	
Black	1.22 (0.99-1.51)	
Hispanic	0.95 (0.78-1.16)	
Asian	1.41 (1.00-1.99)	
Other	1.01 (0.61-1.68)	
Native MELD at LT, per point	1.03 (1.02-1.05)	<0.001
Kidney disease severity		<0.001
On dialysis	Reference	
eGFR <30mL/min/1.73m^2*	0.39 (0.33-0.47)	
eGFR ≥30mL/min/1.73m^2*	0.31 (0.24-0.39)	
Ascites		0.007
None	Reference	
Mild	0.81 (0.65-1.01)	
Moderate-severe	0.70 (0.56-0.88)	
Encephalopathy		0.565
None	Reference	
Grade 1-2	1.10 (0.92-1.32)	
Grade 3-4	1.09 (0.84-1.41)	
Recipient location		0.001
Home	Reference	
Inpatient	0.92 (0.75-1.12)	
ICU	1.36 (1.09-1.70)	
Recipient diabetes	0.94 (0.80-1.11)	0.487
Recipient kidney disease		0.229
Glomerular disease	Reference	
Diabetes	1.27 (0.92-1.74)	
Hypertension	1.17 (0.82-1.67)	
Polycystic kidney disease	0.79 (0.52-1.20)	
Hepatorenal syndrome	1.08 (0.81-1.46)	
Other	1.05 (0.78-1.41)	
Donor age, per year	1.01 (1.01-1.02)	<0.001
Female donor sex	1.04 (0.90-1.22)	0.601
Donor race/ethnicity		0.447
White	Reference	
Black	0.91 (0.74-1.11)	
Hispanic	0.95 (0.78-1.16)	
Asian/other	0.77 (0.55-1.20)	
Donor hypertension	1.35 (1.13-1.61)	0.001
Donor diabetes	1.39 (1.03-1.89)	0.032

DCD donor	2.64 (1.98-3.52)	<0.001
Kidney graft CIT, per hour	1.02 (1.01-1.03)	<0.001
Split liver	0.65 (0.33-1.28)	0.211
Share type		0.020
Local	Reference	
Regional	1.20 (1.01-1.42)	
National	1.55 (1.03-2.36)	
Donor HCV Ab positive	0.92 (0.69-1.22)	0.513
Donor terminal creatinine, per 1mg/dL	1.28 (1.17-1.40)	<0.001
Donor BMI, per kg/m^2	1.02 (1.01-1.03)	0.001
Donor cause of death		0.077
Anoxia	Reference	
CVA	1.22 (1.00-1.48)	
Head trauma	0.92 (0.77-1.10)	
CNS tumor	0.75 (0.27-2.12)	
Other	1.01 (0.65-1.57)	
Transplant era		<0.001
2005-2009	Reference	
2010-2014	1.59 (1.28-1.99)	
2015-2020	1.96 (1.56-2.46)	

<u>Supplemental Table 3:</u> Multivariable logistic regression model evaluating center MP use as a predictor of delayed graft function (N=5,986)

	Covariate-adjusted OR for DGF (95% CI)	p-value
**Center MP use, per 10% increase**	**0.93 (0.90-0.96)**	**<0.001**
Recipient female sex	1.07 (0.94-1.23)	0.299
Recipient age, per year	1.00 (0.99-1.00)	0.483
Recipient race/ethnicity		0.006
White	Reference	
Black	1.19 (0.98-1.45)	
Hispanic	1.17 (0.99-1.39)	
Asian	1.75 (1.26-1.39)	
Other	1.16 (0.72-1.88)	
Native MELD at LT, per point	1.03 (1.02-1.05)	<0.001
Kidney disease severity		<0.001
On dialysis	Reference	
eGFR <30mL/min/1.73m^2*	0.36 (0.30-0.42)	
eGFR ≥30mL/min/1.73m^2*	0.32 (0.25-0.40)	
Ascites		0.004
None	Reference	
Mild	0.79 (0.64-0.96)	
Moderate-severe	0.70 (057-0.86)	
Encephalopathy		0.409
None	Reference	
Grade 1-2	1.05 (0.89-1.23)	
Grade 3-4	0.92 (0.73-1.16)	
Recipient location		<0.001
Home	Reference	
Inpatient	0.99 (0.82-.1.19)	
ICU	1.72 (1.40-2.10)	
Recipient diabetes	0.98 (0.84-1.15)	0.839
Recipient kidney disease		0.446
Glomerular disease	Reference	
Diabetes	1.13 (0.84-1.53)	
Hypertension	1.00 (0.71-1.41)	
Polycystic kidney disease	0.85 (0.58-1.26)	
Hepatorenal syndrome	1.12 (0.85-1.49)	
Other	0.97 (0.74-1.29)	
Donor age, per year	1.01 (1.01-1.02)	<0.001
Female donor sex	1.00 (0.87-1.15)	0.981
Donor race/ethnicity		0.289
White	Reference	
Black	0.88 (0.73-1.07)	
Hispanic	1.11 (0.93-1.33)	
Asian/other	0.97 (0.70-1.34)	
Donor hypertension	1.25 (1.05-1.48)	0.011
Donor diabetes	1.41 (1.06-1.89)	0.018
DCD donor	2.70 (2.07 (3.51)	<0.001
Kidney graft CIT, per hour	1.02 (1.01-1.03)	<0.001

Split liver	0.80 (0.42-1.54)	0.505
Share type		0.001
Local	Reference	
Regional	1.24 (1.06-1.45)	
National	1.84 (1.25-2.71)	
Donor HCV Ab positive	0.91 (0.70-1.19)	0.514
Donor terminal creatinine, per 1mg/dL	1.27 (1.16-1.38)	<0.001
Donor BMI, per kg/m^2	1.02 (1.01-1.03)	0.001
Donor cause of death		0.057
Anoxia	Reference	
CVA	1.21 (1.01-1.45)	
Head trauma	0.91 (0.78-1.08)	
CNS tumor	0.81 (0.30-2.16)	
Other	0.98 (0.64-1.49)	
Transplant era		<0.001
2005-2009	Reference	
2010-2014	1.40 (1.14-1.72)	
2015-2020	1.63 (1.32-2.01)	

Supplemental Table 4: Mixed effects multivariable logistic regression model evaluating kidney allograft preservation strategy as a predictor of primary non-function (N=5,776)

	Covariate-adjusted OR for PNF (95% CI)	p-value
**Kidney allograft preservation**		**0.637**
**Cold storage**	**Reference**	
**Machine perfusion**	**0.88 (0.52-1.49)**	
Kidney disease severity		0.002
On dialysis	Reference	
eGFR <30mL/min/1.73m^2*	0.67 (0.42-1.05)	
eGFR ≥30mL/min/1.73m^2*	0.18 (0.07-0.50)	
Recipient location		0.011
Home	Reference	
Inpatient	0.79 (0.48-1.30)	
ICU	1.72 (1.11-2.66)	
Donor age, per year	1.03 (1.01-1.04)	<0.001
Donor BMI, per kg/m^2	1.05 (1.02-1.08)	0.002
Donor cause of death		0.021
Anoxia	Reference	
CVA	2.01 (1.19-3.40)	
Head trauma	1.48 (0.87-2.52)	
CNS tumor	6.06 (1.66-22.08)	
Other	1.66 (0.49-5.69)	

Supplemental Table 5: Multivariable logistic regression model evaluating center MP use as a predictor of delayed graft function (N=5,986)

	Covariate-adjusted OR for DGF (95% CI)	p-value
**Center MP use, per 10% increase**	**0.94 (0.85-1.04)**	**0.233**
Kidney disease severity		0.002
On dialysis	Reference	
eGFR <30mL/min/1.73m^2*	0.67 (0.43-1.05)	
eGFR ≥30mL/min/1.73m^2*	0.18 (0.07-0.50)	
Recipient location		0.012
Home	Reference	
Inpatient	0.79 (0.48-1.30)	
ICU	1.72 (1.11-2.66)	
Donor age, per year	1.03 (1.01-1.04)	<0.001
Donor BMI, per kg/m^2	1.05 (1.02-1.08)	0.002
Donor cause of death		0.021
Anoxia	Reference	
CVA	2.01 (1.19-3.39)	
Head trauma	1.48 (0.87-2.52)	
CNS tumor	6.11 (1.68-22.2)	
Other	1.65 (0.48-5.64)	

Note: Likelihood ratio test for mixed-effects versus standard logistic regression p=0.074

Supplemental Table 6: Multivariable Cox proportional hazards model evaluating kidney allograft preservation strategy as a predictor of kidney graft survival (N=5,928)

	Covariate-adjusted HR for graft survival (95% CI)	p-value
**Kidney allograft preservation**		**0.230**
**Cold storage**	**Reference**	
**Machine perfusion**	**0.91 (0.78-1.06)**	
Recipient female sex	0.93 (0.83-1.03)	0.167
Recipient age, per year	1.01 (1.00-1.02)	<0.001
Recipient race/ethnicity		0.001
White	Reference	
Black	1.19 (1.03-1.37)	
Hispanic	0.83 (0.72-0.96)	
Asian	1.01 (0.78-1.31)	
Other	0.55 (0.33-0.93)	
Native MELD at LT, per point	1.00 (0.99-1.01)	0.549
Kidney disease severity		0.003
On dialysis	Reference	
eGFR <30mL/min/1.73m^2*	0.84 (0.75-0.95)	
eGFR ≥30mL/min/1.73m^2*	0.81 (0.69-0.96)	
Ascites		0.208
None	Reference	
Mild	0.98 (0.84-1.17)	
Moderate-severe	0.90 (0.76-1.06)	
Encephalopathy		0.136
None	Reference	
Grade 1-2	1.03 (0.90-1.17)	
Grade 3-4	1.19 (0.99-1.43)	
Recipient location		0.012
Home	Reference	
Inpatient	1.08 (0.94-1.25)	
ICU	1.28 (1.09-1.51)	
Recipient diabetes	1.23 (1.09-1.51)	0.001
Recipient kidney disease		0.001
Glomerular disease	Reference	
Diabetes	1.17 (0.94-1.45)	
Hypertension	1.20 (0.94-1.52)	
Polycystic kidney disease	0.63 (0.44-0.88)	
Hepatorenal syndrome	0.95 (0.76-1.19)	
Other	1.10 (0.90-1.35)	
Donor age, per year	1.01 (1.01-1.02)	<0.001
Female donor sex	1.03 (0.92-1.14)	0.620
Donor race/ethnicity		0.073
White	Reference	
Black	1.19 (1.04-1.37)	
Hispanic	1.09 (0.94-1.26)	
Asian/other	1.13 (0.87-1.46)	
Donor hypertension	1.17 (1.03-1.33)	0.017
Donor diabetes	1.05 (0.84-1.33)	0.660

DCD donor	1.51 (1.21-1.88)	<0.001
Kidney graft CIT, per hour	1.01 (1.00-1.01)	0.069
Split liver	1.18 (0.74-1.87)	0.489
Share type		0.011
Local	Reference	
Regional	1.19 (1.04-1.35)	
National	1.38 (0.98-1.94)	
Donor HCV Ab positive	0.93 (0.74-1.17)	0.517
Donor terminal creatinine, per 1mg/dL	1.03 (0.97-1.09)	0.307
Donor BMI, per kg/m^2	1.00 (0.99-1.01)	0.422
Donor cause of death		0.446
Anoxia	Reference	
CVA	1.02 (0.88-1.18)	
Head trauma	0.96 (0.84-1.10)	
CNS tumor	1.28 (0.76-2.17)	
Other	0.76 (0.53-1.10)	
Transplant era		<0.001
2005-2009	Reference	
2010-2014	0.79 (0.70-0.90)	
2015-2020	0.75 (0.64-0.89)	

Supplemental Table 7: Multivariable Cox proportional hazards model evaluating kidney allograft preservation strategy as a predictor of kidney graft survival inclusive of the interaction with KDPI category (N=5,928)

	Covariate-adjusted HR for graft survival (95% CI)	p-value
**Kidney allograft preservation**		**0.014**
**Cold storage**	**Reference**	
**Machine perfusion (at KDPI <20%)**	**0.67 (0.49-0.92)**	
**KDPI category (cold storage preservation)**		**<0.001**
**<20%**	**Reference**	
**20-34%**	**1.03 (0.88-1.21)**	
**35-85%**	**1.45 (1.28-1.65)**	
**>85%**	**1.90 (1.49-2.42)**	
**Allograft preservation * KDPI category**		**0.029**
**Machine perfusion * KDPI <20%**	**Reference**	
**Machine perfusion * KDPI 20-34%**	**1.35 (0.86-2.13)**	
**Machine perfusion * KDPI 35-85%**	**1.59 (1.10-2.29)**	
**Machine perfusion * KDPI >85%**	**2.11 (1.21-3.67)**	
Recipient female sex	0.91 (0.82-1.01)	0.088
Recipient age, per year	1.01 (1.01-1.02)	<0.001
Recipient race/ethnicity		0.001
White	Reference	
Black	1.16 (1.01-1.34)	
Hispanic	0.83 (0.72-0.96)	
Asian	1.01 (0.79-1.31)	
Other	0.54 (0.33-0.91)	
Native MELD at LT, per point	1.00 (0.99-1.01)	0.666
Kidney disease severity		0.003
On dialysis	Reference	
eGFR <30mL/min/1.73m^2*	0.84 (0.75-0.95)	
eGFR ≥30mL/min/1.73m^2*	0.81 (0.69-0.95)	
Ascites		0.322
None	Reference	
Mild	0.99 (0.84-1.16)	
Moderate-severe	0.91 (0.77-1.08)	
Encephalopathy		0.276
None	Reference	
Grade 1-2	1.03 (0.91-1.17)	
Grade 3-4	1.15 (0.96-1.38)	
Recipient location		0.011
Home	Reference	
Inpatient	1.08 (0.94-1.24)	
ICU	1.28 (1.09-1.51)	
Recipient diabetes	1.23 (1.09-1.38)	0.001
Recipient kidney disease		0.002
Glomerular disease	Reference	
Diabetes	1.17 (0.94-1.45)	
Hypertension	1.19 (0.93-1.50)	
Polycystic kidney disease	0.63 (0.45-0.89)	

Hepatorenal syndrome<br>Other	0.96 (0.77-1.20)<br>1.11 (0.91-1.35)	
Female donor sex	1.00 (0.90-1.11)	0.999
Kidney graft CIT, per hour	1.01 (1.00-1.01)	0.043
Split liver	1.10 (0.69-1.35)	0.692
Share type<br>Local<br>Regional<br>National	 Reference<br>1.19 (1.04-1.35)<br>1.26 (0.90-1.75)	0.019
Transplant era<br>2005-2009<br>2010-2014<br>2015-2020	 Reference<br>0.79 (0.70-0.90)<br>0.73 (0.62-0.85)	<0.001

Supplemental Table 8: Multivariable Cox proportional hazards model evaluating center MP use as a predictor of kidney graft survival (N=5,930)

	Covariate-adjusted HR for graft survival (95% CI)	p-value
**Center MP use, per 10% increase**	**1.00 (0.98-1.03)**	**0.851**
Recipient female sex	0.93 (0.76-0.94)	0.172
Recipient age, per year	1.01 (1.00-1.02)	0.001
Recipient race/ethnicity		<0.001
White	Reference	
Black	1.19 (1.06-1.35)	
Hispanic	0.83 (0.71-0.97)	
Asian	0.98 (0.74-1.29)	
Other	0.54 (0.33-0.88)	
Native MELD at LT, per point	1.00 (0.99-1.01)	0.444
Kidney disease severity		0.006
On dialysis	Reference	
eGFR <30mL/min/1.73m^2*	0.85 (0.76-0.94)	
eGFR ≥30mL/min/1.73m^2*	0.82 (0.70-0.95)	
Ascites		0.230
None	Reference	
Mild	1.00 (0.87-1.15)	
Moderate-severe	0.92 (0.80-1.05)	
Encephalopathy		0.132
None	Reference	
Grade 1-2	1.04 (0.90-1.20)	
Grade 3-4	1.20 (1.00-1.45)	
Recipient location		0.055
Home	Reference	
Inpatient	1.08 (0.94-1.23)	
ICU	1.26 (1.04-1.52)	
Recipient diabetes	1.24 (1.11-1.38)	<0.001
Recipient kidney disease		<0.001
Glomerular disease	Reference	
Diabetes	1.17 (0.97-1.41)	
Hypertension	1.22 (0.95-1.58)	
Polycystic kidney disease	0.63 (0.48-0.85)	
Hepatorenal syndrome	0.95 (0.75-1.20)	
Other	1.10 (0.92-1.33)	
Donor age, per year	1.01 (1.01-1.02)	<0.001
Female donor sex	1.03 (0.94-1.14)	0.513
Donor race/ethnicity		0.064
White	Reference	
Black	1.20 (1.04-1.37)	
Hispanic	1.07 (0.94-1.22)	
Asian/other	1.10 (0.83-1.46)	
Donor hypertension	1.16 (1.01-1.34)	0.036
Donor diabetes	1.05 (0.83-1.32)	0.700
DCD donor	1.50 (1.21-1.85)	<0.001
Kidney graft CIT, per hour	1.00 (1.00-1.01)	0.019

Split liver	1.16 (0.70-1.90)	0.569
Share type		0.004
Local	Reference	
Regional	1.21 (1.05-1.38)	
National	1.41 (1.03-1.93)	
Donor HCV Ab positive	0.93 (0.74-1.17)	0.516
Donor terminal creatinine, per 1mg/dL	1.03 (0.87-1.09)	0.319
Donor BMI, per kg/m^2	1.00 (0.99-1.01)	0.582
Donor cause of death		0.265
Anoxia	Reference	
CVA	1.01 (0.88-1.16)	
Head trauma	0.96 (0.83-1.10)	
CNS tumor	1.31 (0.69-2.49)	
Other	0.76 (0.55-1.05)	
Transplant era		0.009
2005-2009	Reference	
2010-2014	0.80 (0.69-0.93)	
2015-2020	0.76 (0.62-0.94(	
